# Decanoic acid additively suppresses IL-1β and enables pharmacologic dexamethasone dose-sparing in LPS/ATP-stimulated THP-1 macrophages

**DOI:** 10.1371/journal.pone.0361029

**Published:** 2026-10-22

**Authors:** Melissa A. Hausburg, Kaysie L. Banton, Christopher D. Cassidy, Robert M. Madayag, Carlos H. Palacio, Jason S. Williams, Gregory W. Thomas, Raphael Bar-Or, David Bar-Or

**Affiliations:** 1 Trauma Research, Swedish Medical Center, Englewood, Colorado, United States of America; 2 Trauma Research, Wesley Medical Center, Wichita, Kansas, United States of America; 3 Trauma Research, Lutheran Medical Center, Wheat Ridge, Colorado, United States of America; 4 Trauma Research, South Texas Health System, McAllen, Texas, United States of America; 5 Trauma Services, Swedish Medical Center, Englewood, Colorado, United States of America; 6 Emergency Services, Wesley Medical Center, Wichita, Kansas, United States of America; 7 Trauma Services, Lutheran Medical Center, Wheat Ridge, Colorado, United States of America; 8 Trauma Services, South Texas Health System, McAllen, Texas, United States of America; 9 Department of Molecular Biology, Rocky Vista University, Parker, Colorado, United States of America; University of the Witwatersrand, SOUTH AFRICA

## Abstract

Systemic inflammatory response syndrome, driven by trauma or infection, involves excessive innate immune activation. Dexamethasone (DEX), a synthetic glucocorticoid, suppresses inflammation, although adverse side effects limit its clinical use. The inflammasome, a multiprotein complex that assembles in response to inflammatory stimuli like ATP after lipopolysaccharide (LPS) priming, facilitates caspase-1 cleavage and release of the proinflammatory cytokine, interleukin (IL)-1β. We investigated whether DEX and decanoic acid (DA), a saturated medium-chain fatty acid, inhibited caspase-1 activity and IL-1β release in an LPS/ATP-stimulated THP-1 macrophage model and whether co-treatment would allow for pharmacologic DEX dose-sparing in vitro. PMA-differentiated THP-1macrophages were primed with LPS overnight and stimulated with ATP for 3 h in the presence of DA, DEX, or both. Viability and cytotoxicity were assessed. Caspase-1 activity and IL-1β were quantified, and EC_50_ values were calculated by nonlinear regression. In control conditions, LPS/ATP stimulation induced 5.56 (q1: 3.51 – q3: 6.38) ng/mL of IL-1β release. DA and DEX inhibited IL-1β with full dose-response EC_50_ values of 131.7 μM and 24.29 nM, respectively. DA concentrations exceeding 800 μM decreased cell viability. An 8 x 9-point DA/DEX dose matrix (0–500 μM DA; 0 to 1e4 nM DEX) was used to interrogate drug interaction for IL-1β inhibition and caspase-1 activity. DEX EC_50_ values remained within a narrow 95% confidence interval of 17.5–26.6 nM, regardless of DA background concentration. DA reduced caspase-1 enzymatic activity with a calculated EC_50_ value of ~140 μM; whereas, DEX did not. These data show that DA and DEX act independently. Furthermore, compared to DEX alone, the presence of DA allowed for equivalent in vitro IL-1β suppression at lower DEX concentrations, supporting pharmacologic DEX dose-sparing. Determination of whether this pharmacologic effect translates to clinically meaningful corticosteroid dose reduction requires further study.

## Introduction

Severe trauma and infection can trigger systemic inflammatory response syndrome, characterized by multi-organ failure and pathologically elevated proinflammatory cytokines like TNF and IL-1β [1,2]. Macrophage secretion of IL-1β requires a multiprotein complex, the inflammasome [3]. IL-1β amplifies inflammation by binding neighboring cell receptors and initiating signaling cascades that recruit additional immune cells [4]. Dysregulation of this process drives chronic inflammatory conditions.

### Canonical and non-canonical inflammasome activation

Macrophage inflammasomes are activated through a two-step process initiated by both pathogen- and damage-associated molecular patterns [3]. In the priming phase, PAMPs engage TLRs that activate NF-κB resulting in the transcriptional upregulation of pro-IL-1β, pro-IL-18, and core inflammasome components. Subsequently, DAMPs, such as extracellular ATP or double-stranded DNA released from dying cells, act as activation signals leading to the assembly of the inflammasome complex [3].

In the canonical pathway, NOD-like receptors such as NLRP3 oligomerize and recruit the adaptor protein, ASC, which in turn promotes the activation of caspase-1. Active caspase-1 cleaves pro-IL-1β and pro-IL-18 into their mature, secreted forms [3].

By contrast, the non-canonical inflammasome involves caspase-4 and −5 in humans which directly recognize cytosolic LPS and other intracellular PAMPs or DAMPs [5]. This interaction induces the cleavage of gasdermin D, generating an N-terminal fragment that forms membrane pores and initiates pyroptosis – a lytic, highly inflammatory form of programmed cell death. Pyroptosis results in release of intracellular proinflammatory molecules, including IL-1β, IL-18, and HMGB1, a nuclear DNA-binding protein that functions as an alarmin to further amplify inflammation through TLR4 and RAGE signaling pathways [5]. Inflammasome signaling constitutes a central mechanism linking cellular stress, infection, and tissue injury to downstream inflammation.

### Mechanisms and limitations of glucocorticoids

Glucocorticoids such as DEX are anti-inflammatory therapies that act through both genomic and non-genomic mechanisms. After diffusing across the plasma membrane, DEX binds cytosolic glucocorticoid receptors, inducing a conformational change that releases the receptor from chaperone proteins [6]. The ligand bound–GR complex then translocates to the nucleus, where it modulates gene expression through direct binding to GRE elements in DNA or by inhibiting proinflammatory transcription factor activity [7]. Together, these mechanisms activate anti-inflammatory genes and inhibit pro-inflammatory gene expression [6].

Despite their robust efficacy, chronic glucocorticoid exposure disrupts normal metabolic and endocrine balance causing immunosuppression and other associated conditions [8]. Many of these toxicities result from broad genomic influence, which extends beyond the regulation of inflammatory mediators [9]. In the context of inflammatory diseases, these systemic adverse effects significantly restrict long-term or repeated use. Consequently, strategies that retain the anti-inflammatory potency of DEX while minimizing systemic exposure are translationally important.

### Rationale for combining dexamethasone and decanoic acid

Evidence indicates that metabolic and lipid mediators can modulate inflammatory signaling pathways that are independent of classical glucocorticoid pathways. Among these, decanoic acid (DA), a 10-carbon medium-chain fatty acid, has gained attention for its capacity to influence membrane dynamics, nuclear receptor signaling, and cytokine release [10–13]. In neurobiology, DA has long been used as a dietary additive in the treatment of refractory epilepsy and shows benefits in other neurological disorders through its ability to decrease oxidative stress, inflammation, and pain [14–17].

Given the potential complementary mechanisms, co-administration of DEX with DA was tested as a pharmacologic strategy to determine whether an analogous level of IL-1β suppression could be achieved at a lower DEX concentration compared to DEX alone.

## Materials and methods

### Dexamethasone and decanoic acid

Dexamethasone Sodium Phosphate Injection, preservative free (product code: 500601 NDC 63323-506-01) was obtained from the in-hospital pharmacy. Decanoic acid sodium salt (CAS RN: 1002-62-6; product no. D0024) was purchased from Tokyo Chemical Industry Co., Ltd.

### THP-1 cell culture

THP1-HMGB1-Lucia cells (thp-gb1lc) were purchased from InvivoGen and grown according to manufacturer’s specifications with normocin (ant-nr-05) and zeocin (ant-zn-05). Cell culture reagents purchased from Thermo Scientific: RPMI 1640 medium (22400105), Fetal Bovine Serum, heat inactivated (A5256801), and Penicillin-Streptomycin (15140122).

### Inflammasome activation and macrophage differentiation

Macrophage differentiation and inflammasome activation procedures were performed in growth medium without the addition of normocin and zeocin. THP1-HMGB1-Lucia cells were plated at 2 x 10^5^ cells per well in black-walled 96-well plates (BRAND plates; cellGrade plus, Ref: 782031) and for full dose response curves, cells were differentiated into macrophages by stimulating with 50 ng/ml PMA for 3h (Sigma; P1585) followed by a 5-day rest period. For dose-matrix experiments, 1.8 x 10^5^ cells per well were differentiated into macrophages by stimulating the cells for 72h with 10 ng/ml PMA followed by a 24h rest period. Cell medium was replaced with control medium or treatments and immediately stimulated overnight with 1 μg/ml LPS-EK (E. coli K12) (InvivoGen; tlrl-eklps). Media was then replaced with fresh control medium or treatments followed by stimulation with 5 mM ATP (InvivoGen; tlrl-atpl) for 3h. Cell culture media was collected following the second hit ATP stimulation, spun to remove any cellular debris, and stored at −80°C until analysis.

### Cell viability/toxicity assay

In the full dose response experiments, cell viability and toxicity were measured using the MultiTox-Fluor Multiplex Cytotoxicity Assay (Promega, G9200) by replacing medium with 1X reagent and incubating the cells for 30–40 min at 37°C. The live-cell protease substrate was Gly-Phe-7-amino-4 trifluoromethyl coumarin (GF-AFC), and the dead-cell protease substrate was bis-(Ala-Ala-Phe)-rhodamine 110 (bis-AAF-R110). Live- and dead-cell protease activity were measured through quantification of fluorescence at 505 and 520 nm with a SpectraMax M2 (Molecular Devices). For the DA/DEX dose matrix experiments, viability was assessed with CellTiter-Fluor Cell Viability Assay (Promega, G6081) and fluorescence was quantified at 505 nm with a SpectraMax i3X Multi-mode microplate reader (Molecular Devices).

### Quantification of IL-1β

For the full dose response experiments, IL-1β was quantified in cell culture media using the human IL-1β ProQuantum Immunoassay from Thermo Fisher Scientific (A355745) according to the manufacturer’s protocol where the media was diluted 1:10 and ligation probes were quantified with a Roche 480 Lightcycler. ProQuantum software was used to perform 5-parameter standard curve fitting and sample IL-1β quantification. For the dose matrix experiments, IL-1β was quantified using a HTRF High-Performance Human IL-1β Detection Kit (Revvity, 62HIL1B2PET) and the FRET signal was detected using a HTRF detection cartridge and a SpectraMax i3X Multi-mode microplate reader (Molecular Devices).

### Quantification of HMGB1-lucia luciferase activity

For the full dose response curves, levels of HMGB1-lucia released into the media following the second hit 3h ATP stimulation were quantified using a Promega GloMax luminometer with the QUANTI-Luc™ 4 Lucia/Gaussia assay (InvivoGen; rep-qlc4lg1) according to the manufacturer’s specifications with the exception that the integration time was extended to 0.5 seconds.

### Quantification of Caspase-1 Activity

Caspase-1 cleavage activity was quantified in media 3h following ATP stimulation using Caspase-Glo 1 inflammasome assays (Promega, G9952), and the manufacturer’s protocol was followed to detect caspase-1 specific activity by subtracting the luciferase signal obtained from samples treated with the caspase-1 specific inhibitor, Ac-YVAD-CHO at a final concentration of 1 μM. Luciferase activity was detected with an injector cartridge in a SpectraMax i3X Multi-mode microplate reader (Molecular Devices).

### Statistical analysis

Data exploration and analyses were done in RStudio [18] with the R programming language [19] using tidyverse, drc, and rstatix packages [20–22]. Normality distribution was assessed using Shapiro-Wilk tests, Q-Q plots, and density plots, and homogeneity of variance was evaluated using Levene’s test. For non-parametric variables, Kruskal-Wallis tests were used to detect overall differences followed by Dunn’s post-hoc tests with an FDR correction for multiple comparisons. One-way ANOVA with Tukey HSD post-hoc tests with FDR correction were performed for normally distributed variables. Percent inhibition values were analyzed using Wilcoxon tests against a reference value of zero, with FDR adjustment for multiple comparisons. Dose-response curve analyses for Figs 1 and 2 were done in Prism (GraphPad) and all other graphs were made with ggplot2 and ggpubr [23,24]. Dose-matrix concentration-response curves were fit using the drc package [22]. Statistical significance was defined as adjusted p (q) < 0.05, with additional thresholds noted as: * q < 0.05, ** q < 0.01, ***q < 0.001.

**Fig 1:**
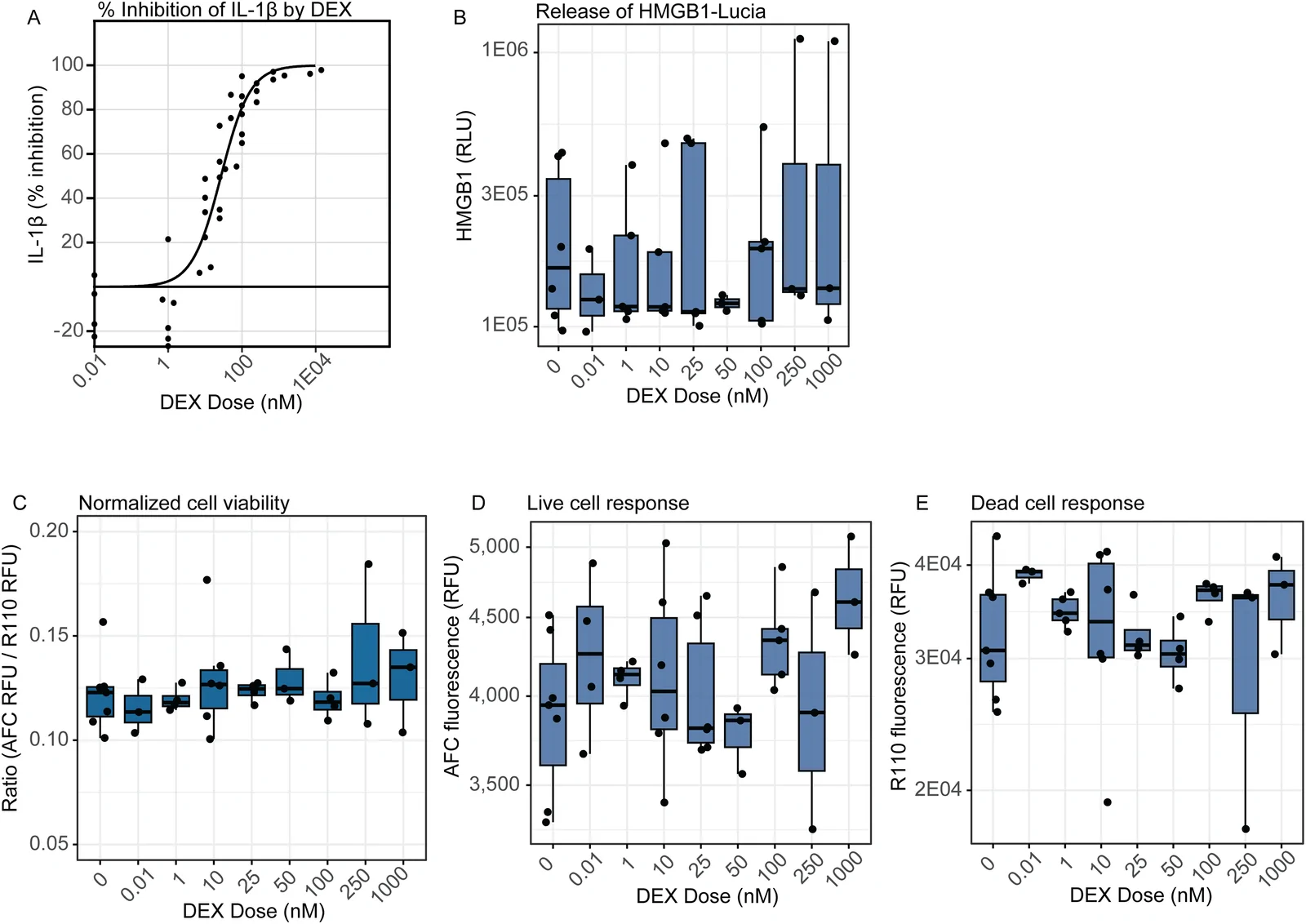
Dose-dependent effects of dexamethasone on IL-1β production and cellular parameters. LPS/ATP-stimulated THP-1 macrophages were treated with increasing concentrations of DEX (0.01-1E04 nM) and assessed for IL-1β release and cellular endpoints. (A) IL-1β inhibition shown as percent inhibition with dose concentrations log_10_ transformed. (B-D) Y-axis is log_10_ scale. (B) HMGB1 release across DEX concentrations, showing no consistent dose-dependent pattern. (C) Normalized cell viability across DEX concentrations shows no evidence of concentration-dependent toxicity. (D) Live-cell protease activity. (E) Dead-cell protease activity. RLU – as a readout of HMGB1-lucia activity. Data shown as individual points representing experimental replicates from 3 to 7 independent experiments (S1 and S2 Tables). Box plots show median and interquartile ranges. Live-cell protease substrate: Gly-Phe-7-amino-4 trifluoromethyl coumarin (AFC); dead-cell protease substrate: bis-(Ala-Ala-Phe)-rhodamine 110 (R110); RFU – relative fluorescent units.

**Fig 2:**
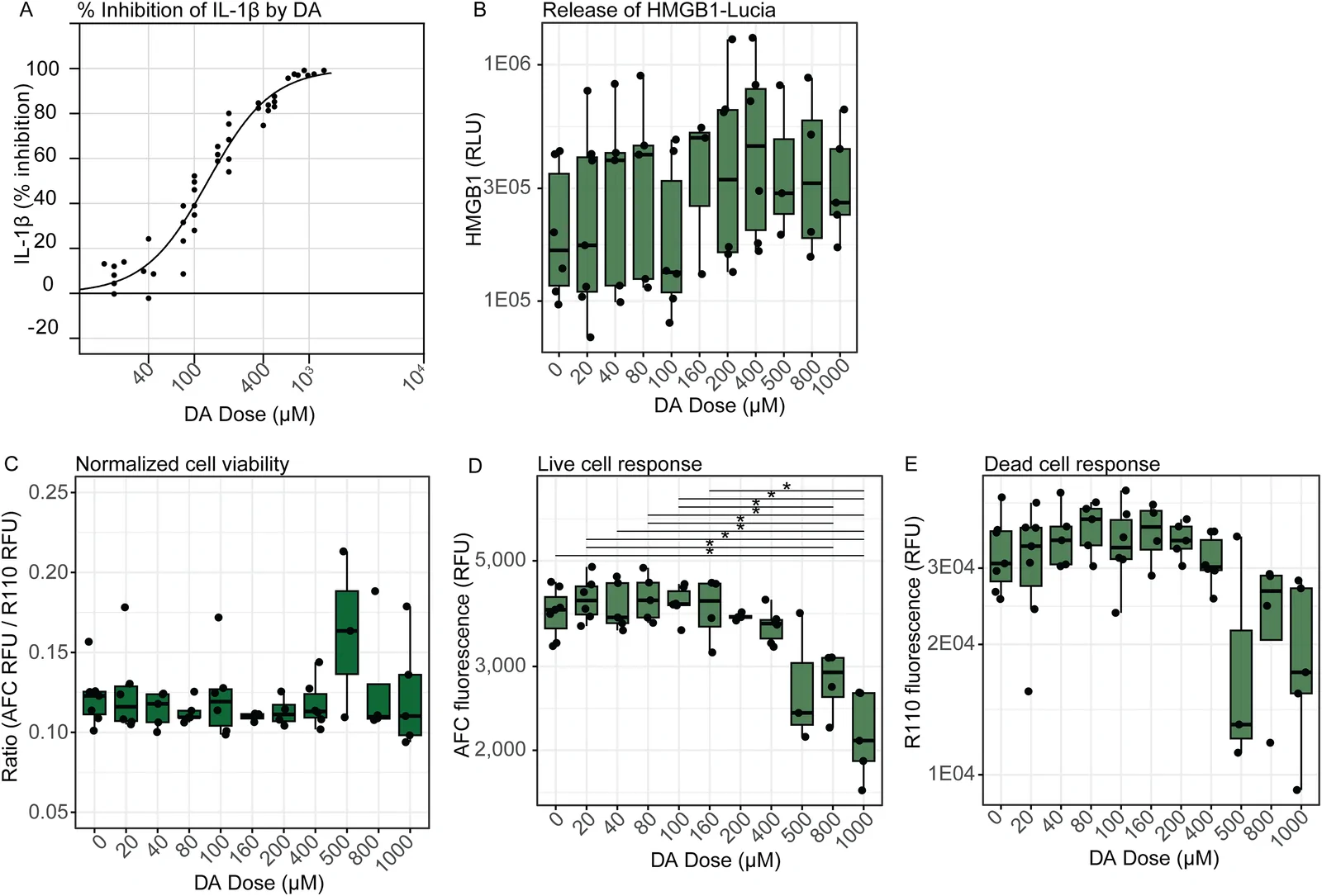
Dose-dependent effects of decanoic acid on IL-1β production and cellular parameters. LPS/ATP-stimulated THP-1 macrophages were treated with increasing concentrations of DA (0-1000 µM) and evaluated for IL-1β release and cellular endpoints. (A) IL-1β inhibition shown as percent inhibition with dose concentrations log_10_ transformed. (B-D) Y-axis is log_10_ scale. (B) HMGB1 release showing high levels of variability with a slight upward trend as DA concentration increases. (C) Normalized cell viability. (D) Live-cell protease activity demonstrating dose-dependent decline at concentrations above 500 μM, with significant reductions at 800 μM and 1000 μM. (E) Dead-cell protease shows a decreasing trend at higher DA concentrations. RLU – as a readout of HMGB1-lucia activity. Data shown as individual points representing replicates from 3 to 7 independent experiments (S1 and S2 Tables). Box plots show median and interquartile ranges; q* < 0.05). Live-cell protease substrate: Gly-Phe-7-amino-4 trifluoromethyl coumarin (AFC); dead-cell protease substrate: bis-(Ala-Ala-Phe)-rhodamine 110 (R110); RFU – relative fluorescent units.

## Results

### Dose-dependent inhibition of IL-1β by dexamethasone and decanoic acid

To evaluate the anti-inflammatory effects of DEX and DA in the LPS/ATP-stimulated THP-1 macrophage model, we performed comprehensive dose-response studies measuring IL-1β production, HMGB1 release, cell viability, and cytotoxicity in inflammasome-stimulated THP-1 cells.

Dexamethasone demonstrated robust dose-dependent inhibition of IL-1β production across a concentration range of 0.01 nM to 1E04 nM (Fig 1A). The dose-response curve of IL-1β normalized as % inhibition over media control showed progressive inhibition with DEX doses >1 nM, with near-complete inhibition at concentrations >100 nM. Curve fit analysis yielded an EC_50_ of 24.29 nM DEX (95% CI: 18.52–31.55 nM) with a Hill slope of 1.018 and an R^2^ of 0.8846, indicating good model fit. Total IL-1β magnitude varied between each experimental replicate and the resulting variability hindered curve fit analysis of raw IL-1β data and showed poor modeling (95% CI: 11.37 to 759.7 nM) with an R^2^ of 0.7657 (S1A Fig); regardless, the estimated EC_50_ of 23.36 nM DEX was consistent with the normalized percent inhibition curves.

Pairwise comparison showed that IL-1β release was significantly inhibited by cells treated with 100nM DEX (median IL-1β pg/ml (q1-q3); 718.56 (571–1493)) compared to control (5559.32 (3509–6383)) and 1 nM DEX (5326.42 (4412–7291)) and by 1000 nM DEX compared to control, 0.01 nM DEX (4943.52 (4710–5341)), and 1 nM DEX (q < 0.05; S1B Fig).

In contrast to its potent effects on IL-1β, DEX had minimal impact on other cellular parameters across the entire dose range. Release of the fusion protein HMGB1-Lucia showed considerable variability due to differences in release between independent experiments but no consistent dose-dependent pattern across all concentrations tested (Fig 1B). Normalized cell viability (Fig 1C), calculated as the ratio of live-cell protease (Fig 1D) to dead-cell protease (Fig 1E), indicated that DEX maintained cellular health across the entire dose range with no evidence of concentration-dependent toxicity.

Decanoic acid exhibited a distinct dose-response profile of IL-1β inhibition compared to DEX. DA demonstrated concentration-dependent inhibition of IL-1β across a range of 20 μM to 1000 μM, with an EC_50_ of 131.7 μM DA (95% CI: 120.0–144.5 μM) and a Hill slope of 1.573, with an R² of 0.9490 (Fig 2A). The inhibition curve showed minimal effect below 100 μM, progressive inhibition between 100–500 μM, and near-complete inhibition at 1000 μM. Similar to the DEX dose analysis, modeling of raw IL-1β values from DA treated cells was hindered by differences in relative IL-1β release between experiments, yielding a poor model fit with an EC_50_ of 164.4 μM DA (95% CI: n/a-254.9 μM) and an R^2^ value of 0.7281 (S1C Fig). As summarized in S1 Table, pairwise comparisons between control and low-mid dose DA showed significant inhibition of IL-1β in groups treated with above 400 μM DA (q < 0.05; control, 5559.32 (3509–6383); 20 μM DA 4783.32 (3159–5787); 40 μM DA, 4014.2 (3246–4654); 80 μM DA, 3463.01 (3064–3755); 100 μM DA, 2287.19 (1809–3756); 400 μM DA, 964.65 (730–1143); 500 μM DA, 540.52 (535–697); 800 μM DA, 146.47 (109–211); 1000 μM DA, 57.59 (54–83); S1 Table and S1D Fig).

In DA treated cells, HMGB1-Lucia levels showed high levels of variability due to differences between each independent experiment, but the trends were consistent, showing a slight upward trend as DA concentration increased compared to vehicle controls (Fig 2B). In contrast to DEX, DA treatment caused a more complex and concentration-dependent effect on cellular health. Normalized cell viability (Fig 2C), calculated as the ratio of live-cell protease (Fig 2D) to dead-cell protease (Fig 2E), appeared to be consistent across treatments; however, upon further inspection, increasing concentrations of DA showed a dose-dependent reduction in the live-cell response at concentrations above 500 μM, with statistically significant reductions observed at 1000 μM DA (2096.45 (1900–2638)) compared to the control (3946.37 (3614–4203) and 20 μM DA (3965.16 (3735–4379)); 40 μM DA (3803.14 (3698–4489)); 80 μM DA (4132.3 (3801–4500)); 100 μM DA (4047.54 (3803–4235)); and 160 μM DA (4133.12 (3646–4480)), and 800 (2923.1 (2597–3129)) versus 20, 80, and 100 μM (S2 Table and Fig 2D). Dead-cell protease activity trended lower at higher DA concentrations, which may indicate an earlier loss of cells due to media exchange or cell death (Fig 2E).

The concentrations associated with the highest loss of cells (>800 μM) were excluded from the subsequent DA/DEX dose-response matrix, which was limited to 0–500 μM DA.

### Combination DEX + DA demonstrates additive inhibition of IL-1β

Given the distinct dose-response profiles of DEX and DA, the potential for complementary mechanisms of action, and an initial single point DEX + DA Bliss independence test showing preliminary evidence of super-additivity, we investigated whether combining DEX and DA could achieve enhanced IL-1β inhibition. To rigorously test this hypothesis, we performed experiments with an 8 x 9-point DA/DEX dose matrix (0–500 μM DA; 0 to 1E04 nM DEX; S2A Fig). We sought to reduce experimental variability by optimizing the stimulation protocol. In contrast to the shorter 3-hour PMA stimulation followed by a 5-day rest period used in our initial dose-response experiments, we transitioned to a 72-hour stimulation period (10 ng/mL PMA), with a 24-hour rest period in fresh media.

Across the full matrix, increasing DA raised the lower plateau, the DA-alone inhibition of IL-1β “floor”, of each DEX dose–response curve (Fig 3A). DEX + DA (0–200 μM) showed sufficient dynamic range for modeling, and to determine whether DA influences the potency of DEX, we compared two non-linear regression models. Model 1 constrained the EC_50_ of DEX to be shared across all concentrations of DA, consistent with a model of independent drug action. To measure any leftward shifts in DEX potency, model 2 permitted DEX EC_50_ values to vary for each DA background concentration. Statistical comparison showed that for IL-1β suppression a shared DEX EC_50_ of 21.7 nM (95% CI 17.5–26.6 nM) fit the data as well as the model permitting separate EC_50_ values for each DA background (EC_50_ Invariance F-Test, F (5,148) = 0.85; p = 0.5153; Fig 3B, top; S3 Table). In parallel, the DA-alone inhibition floor rose with each increased dose, from approximately 0% to 49% across 0–200 µM DA (Fig 3B, bottom). These results empirically demonstrated that DA does not alter DEX mechanistically, and invariant DEX potency together with a rising floor is the quantitative signature of additivity. Comparison of the observed empirical IL-1β percent inhibition against a theoretical predicted additive (independent-action) model demonstrated near perfect concordance across all 72 measured combinations with R^2^ = 0.991 and RMSE 2.4% (Fig 3C; S2B Fig), with no evidence of synergy or antagonism. Single-agent DA EC_50_ values ranged from 130 to 289 µM across experiments (S2C Fig), and the DA dose–response curves essentially retained their form at every DEX background (S2D Fig). The full-matrix analysis therefore did not support the preliminary Bliss-based impression of super-additivity; instead, the data showed that DEX and DA combined additively, consistent with action through independent molecular targets.

**Fig 3:**
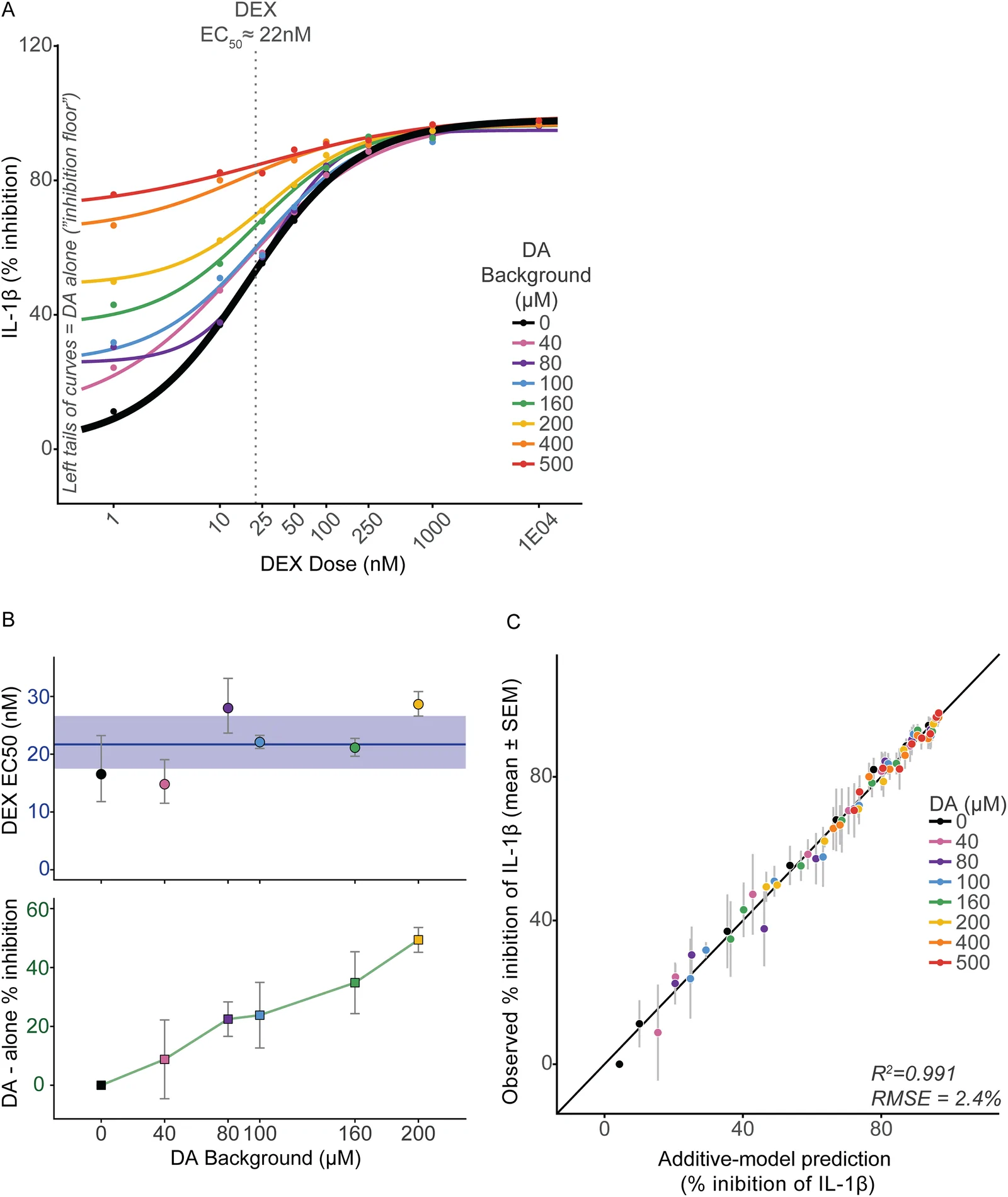
Decanoic acid and dexamethasone inhibit IL-1β release through independent, additive actions. Percent inhibition of IL-1β across an 8 × 9 DA (0–500 µM) × DEX (0–10 µM) matrix; mean of three experiments; color denotes DA dose (black = DEX-alone reference). (A) DEX dose–response at each DA background, increasing DA raises each curve’s floor (DA-alone effect) while the common midpoint (DEX EC₅₀ ≈ 22 nM) is preserved. B) Two stacked sub-panels sharing the DA axis, markers colored by DA (mean ± SEM, n = 3). Top: per-background DEX EC₅₀ (geometric mean, log-scale SEM) scatters around the shared estimate (21.7 nM; line, with 95% CI 17.5–26.6 nM shaded). Bottom: the DA-alone inhibition floor (response at DEX = 0; mean ± SEM) rises with DA dose. (C) Observed vs additive-model prediction for all 72 combinations (R² = 0.991, RMSE 2.4%).

### Decanoic acid, but not dexamethasone, reduces caspase-1 activity

Cleavage of pro-IL-1β to the mature, secreted cytokine requires active caspase-1 proteolytic activity; therefore, we quantified caspase-1 enzymatic activity across the same DA × DEX matrix (Fig 4 and S3A Fig). DA produced dose-dependent reductions in measured caspase-1 activity, and single-agent DA IC_50_ values ranged from 111 to 259 µM across experiments (S3B Fig). Fitting the DA dose–response at each DEX background returned a consistent DA EC₅₀ at low DEX (EC₅₀ ≈ 140 µM at DEX background concentrations < 250nM), with decreasing EC_50_ estimates at the two highest DEX background concentrations (94.7, 78.7 nM). These decreasing EC_50_ values reflect the increased span of those curves rather than a change in DA potency (Fig 4A; S4 Table). The measured caspase-1 surface confirmed this pattern, with inhibition increasing along the DA axis but not the DEX axis (S3A Fig). Line graphs (without curve fits) of DEX dose-response curves with increasing DA background concentrations showed that DEX increased caspase-1 activity (DA = 0; negative inhibition) compared to no drug controls (Fig 4B). This effect was overcome by progressively higher concentrations of DA. These data support an association between DA treatment and reduced caspase-1 activity in this model.

**Fig 4:**
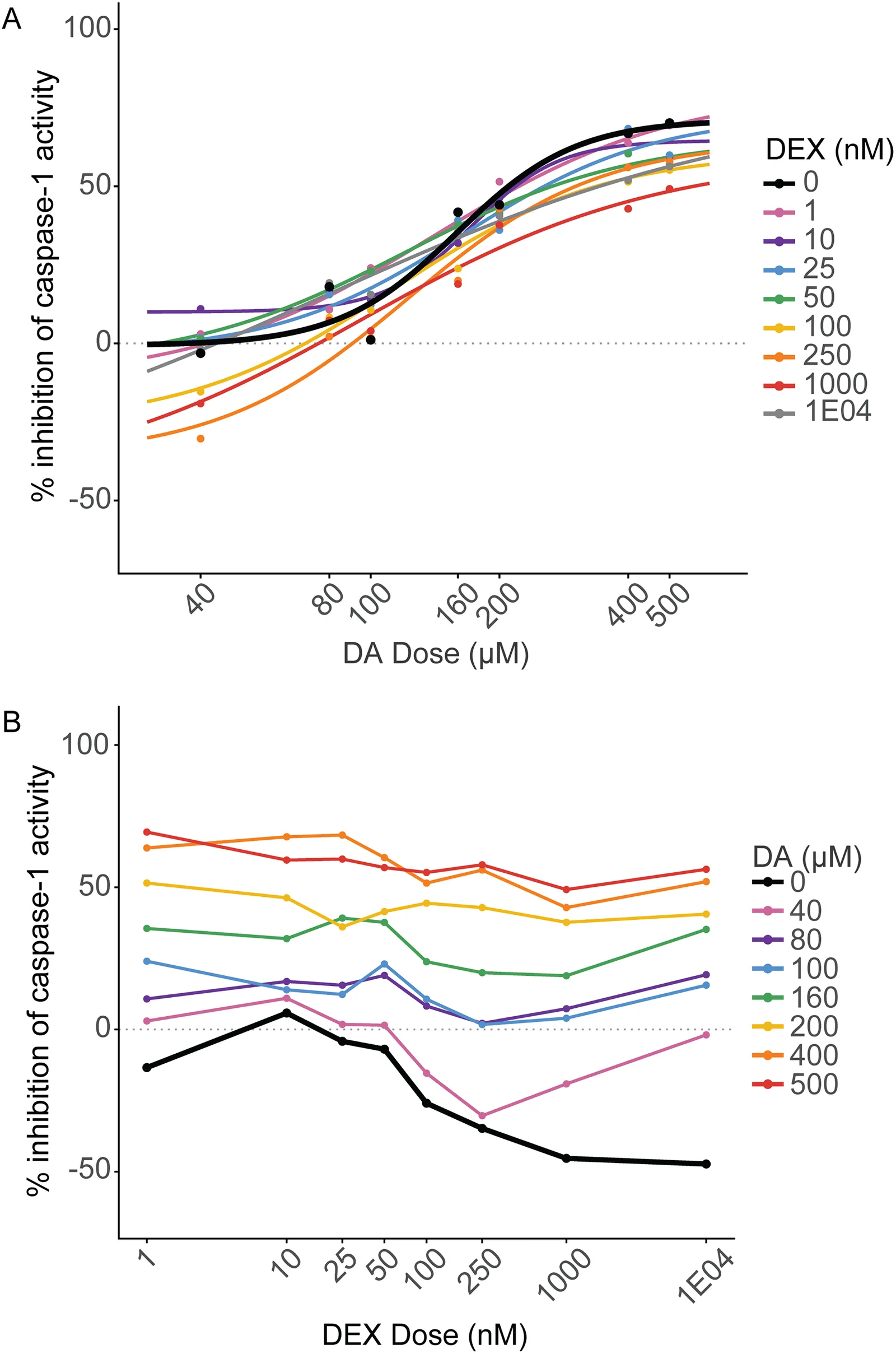
Decanoic acid inhibits caspase-1; dexamethasone does not. Caspase-1 activity as percent inhibition vs the 2-hit–stimulated, drug-free control; plate replicates averaged within experiment, mean of three experiments. (A) DA dose–response fitted (four-parameter log-logistic) at every DEX background, colored by DEX (black = DEX-alone reference, as in Fig 3). Low-DEX-background curves give DA EC_50_ ≈ 140 µM; high-DEX curves start from depressed baselines and shift accordingly, reflecting the DEX-driven increased caspase-1 activity, rather than a change in DA potency. (B) Caspase-1 inhibition vs DEX, one series per DA background, colored by DA. Means are connected directly without a fitted curve; inhibition stacks with DA dose while staying essentially flat across DEX. DEX-alone curves (DA = 0) show increased caspase-1 activity over no-drug control (inhibition becomes negative).

## Discussion

### DEX and DA suppress IL-1β in LPS/ATP-stimulated THP-1 macrophages

This study demonstrates that DEX and DA, individually and in combination, reduce IL-1β release from LPS/ATP-stimulated THP-1 macrophages, and full dose-matrix analyses indicate that DEX + DA interact additively. Moreover, DA reduces measurable caspase-1 enzymatic active, whereas DEX did not.

### Rationale for ATP-induced inflammasome model

Extracellular ATP functions as a canonical DAMP that activates the NLRP3 inflammasome through P2X7 receptor engagement, leading to potassium efflux, mitochondrial depolarization, and subsequent caspase-1 activation culminating in IL-1β and IL-18 release [25–27]. The LPS/ATP-stimulated THP-1 macrophage model employed here provides an experimental system for probing ATP-responsive inflammasome pathways; however, it does not reproduce the cellular heterogeneity or tissue environment that are relevant to systemic or chronic inflammatory diseases.

### Mechanisms of DEX and DA anti-inflammatory activity

Mechanistically, it is known that DEX suppresses IL-1β through GR-mediated transcriptional repression of inflammatory genes, primarily via repression of NF-κB activity, thereby reducing pro-IL-1β transcription [28]. Importantly, DEX maintained cell viability across all tested concentrations and did not alter HMGB1 release, confirming the absence of cytotoxicity under these conditions.

Multiple intrinsic anti-inflammatory mechanisms may contribute to the ability of DA to inhibit IL-1β in this model. Historically characterized as a permeation enhancer for drug delivery across epithelial and endothelial barriers [29,30], and although the precise molecular targets remain to be defined, the known effects of DA on biological membranes suggest potential involvement of membrane-mediated pathways. As a possible reflection of concentration-dependent membrane effects, a decline in live-cell protease activity was observed at the highest DA doses tested. Conversely, at lower concentrations of DA, the live-cell response was intact, and importantly, so was the anti-inflammatory activity.

### Combination pharmacology: effects through independent mechanisms

The full dose-matrix analysis establishes that DEX and DA inhibit IL-1β additively, as DEX potency remained invariant across DA backgrounds (shared EC_50_ ≈ 22 nM), while DA contributed an independent, dose-dependent inhibition floor (Fig 3). The caspase-1 measurements support the pharmacologic separation, since DA was associated reduced caspase-1 enzymatic activity, whereas DEX was not (Fig 4). These data are consistent with the two agents engaging distinct, non-overlapping mechanisms. Established in glucocorticoid biology, DEX suppresses NF-κB-driven transcription of pro-IL-1β at inflammasome priming, and independently, DA exerts its effects at or upstream of the generation of measurable caspase-1 enzymatic activity resulting in additive IL-1β suppression (Fig 5).

**Fig 5:**
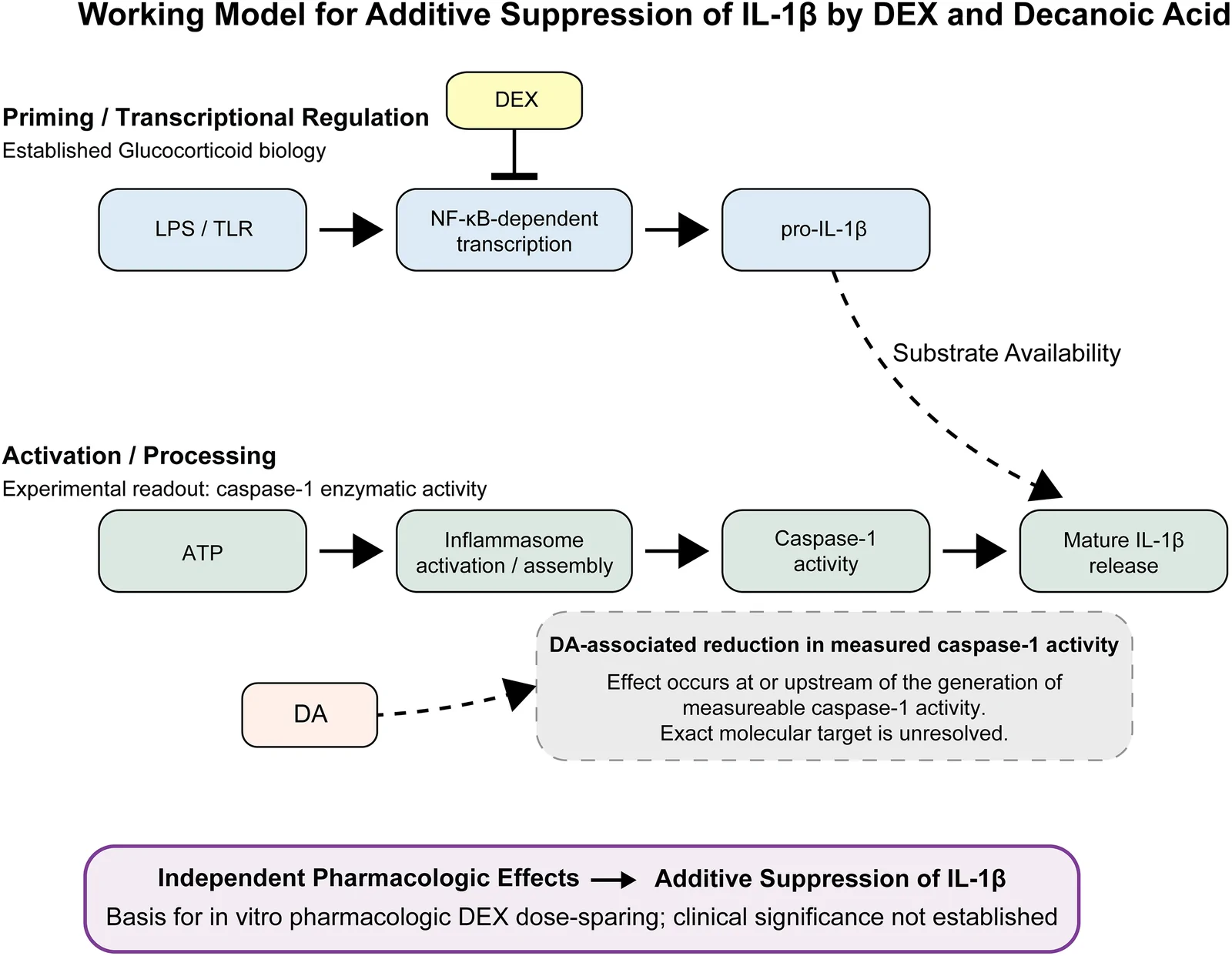
Working model for the additive suppression of IL-1β by DEX and DA. The effect of DEX is shown at the priming/transcriptional level based on established glucocorticoid biology. Independently, DA is associated with reduced levels of measurable caspase-1 enzymatic activity; however, the precise molecular target is unresolved; thus, the model depicts independent pharmacological effects that produce additive IL-1β suppression and an in vitro basis for DEX dose-sparing [31].

### Limitations and future directions

The present study was conducted exclusively in PMA-differentiated THP-1 cells using LPS and ATP stimulation. Confirmation in primary human macrophages and other disease-relevant cell types, and with additional inflammasome stimuli would be required to determine the generalizability and physiological relevance of this response.

Although caspase-1 enzymatic activity was measured, the experiments did not directly evaluate NLRP3 inflammasome assembly, ASC speck formation, or gasdermin D cleavage. Consequently, the molecular target responsible for the DA-associated reduction in caspase-1 activity remains unknown.

We observed a decrease in live-cell responses at the highest concentrations of DA tested; however, DA suppressed IL-1β and caspase-1 enzymatic activity at considerably lower concentrations. These data represent an in vitro separation between efficacy and cell viability rather than an establishing a therapeutic window. Whether comparable tissue concentrations can be achieved safely in vivo is unknown and would require pharmacokinetic and toxicologic evaluation. Further, the observed pharmacologic DEX dose-sparing in vitro does not establish that the magnitude of DEX reduction would be clinically meaningful or would reduce glucocorticoid-associated adverse effects.

### Potential translational relevance

The present findings do not establish therapeutic efficacy but rather provide a rationale for further investigation of DEX + DA combination treatment in disease-relevant experimental systems. Osteoarthritis represents one of the potential contexts for evaluation because IL-1β plays a central role in cartilage matrix degradation, synovial inflammation, and pain sensitization, while NLRP3 inflammasome activation perpetuates the chronic inflammatory state [32–34]. Current intra-articular corticosteroid injections provide patients with transient relief, and repeated administration can exacerbate cartilage loss due to both mechanical and metabolic effects propagated by the steroid [35,36]. Within the context of DEX, dose reduction is clinically meaningful as repeated long-term exposure carries risks of immunosuppression, hyperglycemia, muscle wasting, and bone demineralization [8,37].

In the present study, the additive interaction of DEX and DA demonstrates pharmacologic dose-sparing in vitro since the independent inhibitory contribution of DA permits a given degree of IL-1β suppression at lower DEX concentrations compared to DEX alone. Importantly though, these data do not demonstrate a clinically meaningful steroid dose-sparing effect, which would require in vivo pharmacokinetic/pharmacodynamic studies and ultimately clinical investigation.

An important conceptual outcome of this work is the recognition of excipients as bioactive modulators rather than inert formulation components. DA may exemplify a multifunctional therapeutic excipient that has the potential to enhance drug delivery while contributing independent anti-inflammatory effects [38]. Ongoing clinical trials of DA in oral formulations further support its translational feasibility and regulatory acceptance as a safe, bioactive component [39].

## Conclusions

In summary, DEX + DA additively reduced IL-1β release in LPS/ATP-stimulated THP-1 macrophages, and DA treatment was associated with lower levels of caspase-1 enzymatic activity, whereas DEX treatment was not. These results provide a mechanistic basis for developing DEX + DA combination formulations for intra-articular osteoarthritis therapy and possibly other inflammasome-driven conditions. Validation in primary human cells and relevant animal in vivo models will be crucial for clinical translation.

## Supporting information

S1 Fig Additional analyses of dose-response relationships for DEX and DA. (A, C) Dose-response curves of raw IL-1β (pg/mL) values where dose concentrations were log_10_ transformed. (A, C) Box plots showing median and interquartile ranges of IL-1β values with replicates overlayed at each dose on a log_10_ y-axis (B, D). (A) Curve fit analysis of DEX IL-1β inhibition shows a calculated EC_50_ of 23.36 nM DEX (95% CI: 11.37 to 759.7 nM) and an R^2^ of 0.7657 (B) Dose-dependent inhibition of IL-1β by DEX. (C) Curve fit analysis of DA IL-1β inhibition shows poor model fit with an EC_50_ of 164.4 μM DA (95% CI: n/a-254.9 μM) and an R^2^ value of 0.7281. (D) Dose-dependent inhibition of IL-1β by DA. Data shown as individual points representing experimental replicates from 3 to 7 independent experiments; q: * < 0.05, ** < 0.01, *** < 0.001).
(TIF)

S2 FigDEX and DA inhibit IL-1β through independent, additive actions.(A) Mean percent IL-1β inhibition across the 8x9 DA/DEX dose matrix (n = 3). (B) Observed percentage IL-1β inhibition minus additive-model predicted inhibition; residuals small and unstructured (Root Mean Square Error (RMSE) = 2.4%). (C) 3PL curve fits and IC_50_ estimates per matrix experiment for DA-dependent IL-1β percent inhibition with dose concentrations log_10_ transformed. (D) DA dose-response at each DEX background; 4PL curve fits only where well-fitted. At DEX ≥ 100 nM the response is saturated and unable to be modeled.(TIF)

S3 Fig DA inhibits caspase-1; dexamethasone does not. (A) Mean percent caspase-1 inhibition across the 8x9 DA/DEX dose matrix (n = 3). (B) 3PL curve fits and IC_50_ estimates per matrix experiment for DA-dependent caspase-1 percent inhibition with dose concentrations log_10_ transformed.
(TIF)

S4 Fig Graphical Abstract. In vitro dose-sparing strategy for additive inhibition of IL-1β release in LPS/ATP-stimulated THP-1 macrophages [31]. (TIF)

S1 Table Comparisons of decanoic acid doses showing a significant decrease in IL-1β. (DOCX)

S2 Table Comparison of DA treatment groups that showed significant decreases in cellular viability. (DOCX)

S3 Table EC_50_ Invariance F-Test: Statistical comparison of DEX dose-response models, one shared EC_50_ value or modeled with fits to each DEX dose-response curve with DA background concentrations of 0–200 μM. (DOCX)

S4 Table DA dose-response curve EC_50_ values for caspase-1 inhibition across DEX backgrounds. (DOCX)

S1 Data Raw values for full dose response curves of DA and DEX. (XLSX)

S2 Data Summary showing n, min, max, median, iqr, mean, sd, se, ci for raw values. (CSV)

S3 Data Raw IL-1β values for DA / DEX dose response matrix experiments. (CSV)

S4 Data Raw caspase-1 values for DA / DEX dose response matrix experiments. (CSV)

## Abbreviations

AP-1: activator protein-1
ASC: apoptosis-associated speck-like protein containing a caspase recruitment domain
DA: Decanoic acid; PubChem CID 2969
DAMPs: damage-associated molecular patterns
DEX: Dexamethasone
FDR: false discovery rate
GR: glucocorticoid receptor
GRE: glucocorticoid response element
HMGB1: high mobility group box 1
IL: interleukin
LPS: lipopolysaccharide
NF-κB: nuclear factor kappa-light-chain-enhancer of activated B cells
NOD: nucleotide-binding oligomerization domain
OA: osteoarthritis
PAMPs: pathogen-associated molecular patterns
PGPS: peptidoglycan-polysaccharide
PMA: phorbol 12-myristate 13-acetate
RAGE: receptor for advanced glycation end products
RFU: relative fluorescence units
RLU: relative light units
RMSE: root mean square error
TLR: toll-like receptor
TNF: tumor necrosis factor
q: adjusted p value
