# Supplementary material for: Decanoic acid additively suppresses IL-1β and enables pharmacologic dexamethasone dose-sparing in LPS/ATP-stimulated THP-1 macrophages
Source: PLoS One. 2026 Oct 22;21(10):e0361029. doi: 10.1371/journal.pone.0361029 (PMC13690199; doi:10.1371/journal.pone.0361029)
Supplement: Supplementary file 1 [file pone.0361029.s005.docx]

**S1 Table. Comparisons of decanoic acid doses showing a significant decrease in IL-1β.**

| Treatment | Comparison Treatment | Test Statistic | p value | q value |
| --- | --- | --- | --- | --- |
| Control | vs |  |  |  |
|  | 400 μM DA | -3.43 | 6.05E-04 | 0.006 |
|  | 500 μM DA | -3.06 | 2.25E-03 | 0.012 |
|  | 800 μM DA | -3.89 | 9.90E-05 | 0.002 |
|  | 1000 μM DA | -4.63 | 3.65E-06 | <0.001 |
| 20 μM DA | vs |  |  |  |
|  | 400 μM DA | -3.09 | 2.01E-03 | 0.012 |
|  | 500 μM DA | -2.78 | 5.41E-03 | 0.025 |
|  | 800 μM DA | -3.59 | 3.30E-04 | 0.005 |
|  | 1000 μM DA | -4.31 | 1.66E-05 | <0.001 |
| 40 μM DA | vs |  |  |  |
|  | 800 μM DA | -2.94 | 3.29E-03 | 0.016 |
|  | 1000 μM DA | -3.5 | 4.61E-04 | 0.005 |
| 80 μM DA | vs |  |  |  |
|  | 800 μM DA | -2.6 | 9.36E-03 | 0.037 |
|  | 1000 μM DA | -3.14 | 1.67E-03 | 0.011 |
| 100 μM DA | vs |  |  |  |
|  | 800 μM DA | -2.68 | 7.26E-03 | 0.031 |
|  | 1000 μM DA | -3.34 | 8.48E-04 | 0.007 |
