# Supplementary material for: Decanoic acid additively suppresses IL-1β and enables pharmacologic dexamethasone dose-sparing in LPS/ATP-stimulated THP-1 macrophages
Source: PLoS One. 2026 Oct 22;21(10):e0361029. doi: 10.1371/journal.pone.0361029 (PMC13690199; doi:10.1371/journal.pone.0361029)
Supplement: Supplementary file 2 [file pone.0361029.s006.docx]

**S2 Table. Comparison of DA treatment groups that showed significant decreases in cellular viability.**

| Treatment | Comparison Treatment | Test Statistic | p value | q value |
| --- | --- | --- | --- | --- |
| Control | vs |  |  |  |
|  | 1000 μM DA | -3.07 | 0.002 | 0.029 |
| 20 μM DA | vs |  |  |  |
|  | 800 μM DA | -2.86 | 0.004 | 0.034 |
|  | 1000 μM DA | -3.52 | <0.001 | 0.011 |
| 40 μM DA |  |  |  |  |
|  | 1000 μM DA | -2.9 | 0.004 | 0.034 |
| 80 μM DA | vs |  |  |  |
|  | 800 μM DA | -2.81 | 0.005 | 0.034 |
|  | 1000 μM DA | -3.43 | 0.001 | 0.011 |
| 100 μM DA | vs |  |  |  |
|  | 800 μM DA | -2.8 | 0.005 | 0.034 |
|  | 1000 μM DA | -3.45 | 0.001 | 0.011 |
| 160 μM DA | vs |  |  |  |
|  | 1000 μM DA | -2.77 | 0.006 | 0.034 |
