# Supplementary material for: Decanoic acid additively suppresses IL-1β and enables pharmacologic dexamethasone dose-sparing in LPS/ATP-stimulated THP-1 macrophages
Source: PLoS One. 2026 Oct 22;21(10):e0361029. doi: 10.1371/journal.pone.0361029 (PMC13690199; doi:10.1371/journal.pone.0361029)
Supplement: Supplementary file 3 [file pone.0361029.s007.docx]

**S3 Table. EC_50_ Invariance F-Test: Statistical comparison of DEX dose-response models, one shared EC_50_ value or modeled with fits to each DEX dose-response curve with DA background concentrations of 0 to 200 μM.**

| Model Comparison | Model Description* | Model Degrees of Freedom | Residual Sum of Squares | Degrees of Freedom between Models | F-value | p-value |
| --- | --- | --- | --- | --- | --- | --- |
| Model 1 | Shared EC_50_, Shared Slope | 153 | 10052.6 |  |  |  |
| Model 2 | Free EC_50_, Shared Slope | 148 | 9771.4 | 5 | 0.8517 | 0.5153 |

*Both models were log-logistic 4-parameter
