# Supplementary material for: Decanoic acid additively suppresses IL-1β and enables pharmacologic dexamethasone dose-sparing in LPS/ATP-stimulated THP-1 macrophages
Source: PLoS One. 2026 Oct 22;21(10):e0361029. doi: 10.1371/journal.pone.0361029 (PMC13690199; doi:10.1371/journal.pone.0361029)
Supplement: Supplementary file 4 [file pone.0361029.s008.docx]

**S4 Table. DA dose-response curve EC_50_ values for caspase-1 inhibition across DEX backgrounds**

| DEX background concentration (nM) | EC_50_ | R^2^ | Curve Span % |
| --- | --- | --- | --- |
| 0 | 159.27 | 0.95 | 71.92 |
| 1 | 140.28 | 0.99 | 92.79 |
| 10 | 172.07 | 0.98 | 54.6 |
| 30 | 164.6 | 0.96 | 76.11 |
| 50 | 121.49 | 0.99 | 73.04 |
| 100 | 113.66 | 0.98 | 87.1 |
| 250 | 122.16 | 0.98 | 100.97 |
| 1000 | 94.69 | 0.98 | 105.4 |
| 10000 | 78.73 | 0.99 | 125.2 |
